# *Vibrio vulnificus* RtxA Is a Major Factor Driving Inflammatory T Helper Type 17 Cell Responses *in vitro* and *in vivo*

**DOI:** 10.3389/fimmu.2018.02095

**Published:** 2018-09-19

**Authors:** Arim Lee, Myun Soo Kim, Daeho Cho, Kyung Ku Jang, Sang Ho Choi, Tae Sung Kim

**Affiliations:** ^1^Department of Life Sciences, College of Life Sciences and Biotechnology, Korea University, Seoul, South Korea; ^2^Institute of Convergence Science, Korea University, Seoul, South Korea; ^3^National Research Laboratory of Molecular Microbiology and Toxicology, Department of Agricultural Biotechnology, Seoul National University, Seoul, South Korea

**Keywords:** *V. vulnificus*, RTX toxin, dendritic cells, Th17, mouse

## Abstract

T helper type 17 (Th17) cells are a subset of pro-inflammatory T helper cells that mediate host defense and pathological inflammation. We have previously reported that host dendritic cells (DCs) infected with *Vibrio vulnificus* induce Th17 responses through the production of several pro-inflammatory cytokines, including interleukin (IL)-1β and IL-6. *V. vulnificus* produces RTX toxin (RtxA), an important virulence factor that determines successful pathophysiology. In this study, we investigated the involvement of RtxA from *V. vulnificus* in Th17 cell induction through the activation and maturation of DCs. The increased expression of the DC surface marker CD40 caused by *V. vulnificus* wild-type infection was reduced by *rtxA* gene mutation in *V. vulnificus*. The mRNA and protein levels of Th17 polarization-related cytokines also decreased in *V. vulnificus rtxA* mutant-infected DCs. In addition, the co-culture of Th cells and DCs infected with *rtxA* mutant *V. vulnificus* resulted in reduction in DC-mediated Th17 responses. Th17 cell responses in the small intestinal lamina propria decreased in mice inoculated with *V. vulnificus rtxA* mutant as compared to those inoculated with the wild-type strain. These decreases in DC maturation, Th17-polarizing cytokine secretion, and Th17 responses attributed to *rtxA* mutation were restored following infection with the *rtxA* revertant strain. Furthermore, the mutation in the *hlyU* gene encoding the activator of *rtxA1* gene reproduced the results observed with *rtxA* mutation. Taken together, *V. vulnificus*, by means of RtxA, induces inflammatory Th17 responses, which may be associated with adaptive responses of the host against *V. vulnificus* infection.

## Introduction

*Vibrio vulnificus* is a halophilic estuarine bacterium that causes disease by infection of wounds or ingestion of contaminated seafood (1). *V. vulnificus* is the causative agent of several diseases, including necrotizing fasciitis, gastroenteritis, and primary septicemia.

The pathogenicity of *V. vulnificus* is associated with several virulence factors that include lipopolysaccharide (LPS) (2), capsular polysaccharide (CPS) (3), elastolytic protease (VvpE) (4), hemolysin (VvhA) (5), peroxiredoxin (6), and RTX toxin (RtxA) (7). Of these, the VvhA and RtxA cytotoxins may be the most important virulence factors.

Recent studies have elucidated the adaptive immune response against *V. vulnificus* infection (8, 9). Previously we demonstrated that *V. vulnificus* infection induces Th17 responses via maturation and activation of dendritic cells (DCs). In addition, *V. vulnificus* infection following oral ingestion results in the induction of Th17 cell response in the small intestinal lamina propria (8). Furthermore, *V. vulnificus* infection induces Th1 and T follicular helper (Tfh) cells and VvhA is involved in these responses. (9). However, the specific virulence factor of *V. vulnificus* necessary for the induction of Th17 cells is unclear.

RtxA is a member of the multifunctional-autoprocessing repeats in toxin, a subgroup of RTX toxin family with tandem nonapeptide repeats near the C-terminal region (10). RtxA is exported via the modified type I secretion system (11). Several studies have evaluated the cytotoxic and cytopathic effects of RtxA, and reported that RtxA was related to the *in vivo* growth of bacteria (12), host cell necrosis (12), apoptosis (13), inflammasome activation (14), actin aggregation (15), phagocytosis inhibition (16), and the production of reactive oxygen species (17). The role of RTX toxin in pathogenesis has been investigated in *V. vulnificus* and other bacterial species. However, its effect on host adaptive immune responses against *V. vulnificus* infection remains unclear. Therefore, we investigated whether RtxA influences Th17 cell responses following *V. vulnificus* infection.

We found that the *rtxA* mutant of *V. vulnificus* induced lower levels of DC maturation and activation than wild-type (WT) *V. vulnificus*. Furthermore, the ability of *V. vulnificus* to induce Th17 cell responses became diminished following mutation of the *rtxA* gene, consistent with the observation of reduced expression and secretion of Th17 cell-polarizing cytokines. Involvement of RtxA in Th17 cell induction was confirmed through the recovery of the decreased Th17-related responses following infection with an *rtxA* revertant. Furthermore, the mutation of the *hlyU* gene, an anti-repressor of *rtxA* gene, resulted in defective Th17 cell responses. Taken together, our results suggest that *V. vulnificus* induces Th17 cell responses *in vitro* and *in vivo* through RtxA.

## Materials and methods

### Mice

All animals used in this study were purchased from Orient Bio Inc. (Seoul, Korea). Seven- to 11-week-old female C57BL/6 mice were used for experiments. All mice were capable of accessing a standard laboratory chow diet (cat. no. 1314; Altromin Spezialfutter GmbH & Co. KG, Lage, Nordrhein-Westfalen, Germany) and water. The animals were housed in an SPF facility under a strict light cycle (lights on at 07:00 a.m. and off at 07:00 p.m.) at 22 ± 1°C and 52.5 ± 2.5% relative humidity, and all animal experiments were ethically performed in accordance with the guidelines of the Korea University Institutional Animal Care and Use Committee (Seoul, Korea; approval no. KUIACUC-2016-170, 2017-113).

### Bacterial strains, plasmids, and culture conditions

All *V. vulnificus* strains and plasmids used in this study are listed in Table 1. Unless otherwise noted, *V. vulnificus* strains were cultured in Luria-Bertani (LB) medium supplemented with 2.0% (*wt*/*vol*) sodium chloride (NaCl; LBS) at 30°C. For the infection experiments, the bacteria were cultured overnight, followed by their incubation in fresh LBS medium at 30°C. The culture was diluted to ~1.8 × 10^8^ CFU/mL in LBS and centrifuged at 2,420 × g for 3 min at room temperature. The cells were resuspended in antibiotic-free growth medium before infection of DCs or in phosphate-buffered saline (PBS) before oral administration into mice.

**Table 1:** Bacterial strains and plasmids used in this study.

**Strain or plasmid**	**Relevant characteristics^a^**	**Reference or source**
**BACTERIAL STRAINS**		
***V. vulnificus***
MO6-24/O	Clinical isolate; virulent	(18)
HS03	MO6-24/O with *smcR::nptI*; Km^r^	(19)
OH0701	MO6-24/O with *aphC1::nptI*; Km^r^	(6)
ZW141	MO6-24/O with Δ*hlyU*	(20)
KK1408	MO6-24/O with Δ*vvhBA*	(21)
KK1604	MO6-24/O with Δ*rtxA*::*nptI*; Km^r^	This study
KK1606	MO6-24/O with *rtxA* restored to Km^s^ by recombination with-type sequence	This study
***E. coli***
S17-1 λ*pir*	λ-*pir* lysogen; *thi pro hsdR hsdM^+^ recA* RP4-2 Tc::Mu-Km::Tn7;Tp^r^ Sm^r^; host for π-requiring plasmids; conjugal donor	(22)
**PLASMIDS**		
pGEM-T Easy	PCR product cloning vector; Ap^r^	Promega
pKK1621	pGEM-T easy with Δ*rtxA*; Ap^r^	This study
pKK1625	pGEM-T easy with Δ*rtxA*::*nptI*; Ap^r^, Km^r^	This study
pUC4K	pUC4 with *nptI*; Ap^r^ Km^r^	(23)
pDM4	R6K γ *ori sacB*; suicide vector; *oriT* of RP4; Cm^r^	(24)
pKK1628	pDM4 with Δ*rtxA*::*nptI*; Km^r^	This study
pKK1629	pDM4 with intact *rtxA*	This study

a *Km^r^, kanamycin resistant; Km^s^, kanamycin sensitive; Tp^r^, trimethoprim resistant; Sm^r^, streptomycin resistant; Cm^r^, chloramphenicol resistant; Ap^r^, ampicillin resistant*.

### Construction of the *rtxA* mutant and revertant strain

To inactivate *rtxA* gene *in vitro*, the region from 15- to 1,068-bp downstream of the translational initiation codon of *rtxA* was deleted using the polymerase chain reaction (PCR)-mediated linker-scanning mutation method, as previously described (7, 25). In brief, Briefly, pairs of primers RTXA01-F and -R (for amplification of the 5′ amplicon) or RTXA02-F and -R (for amplification of the 3′ amplicon) were designed and used (Table 2). The resulting *rtxA* mutant was amplified by PCR using the mixture of both amplicons as the template and RTXA01-F and RTXA02-R as primers. The 1.2-kb DNA fragment carrying *nptI* encoding for aminoglycoside 3′-phosphotransferae and conferring resistance to kanamycin (26) was inserted into a unique BamHI site present within the Δ*rtxA* in pKK1621. The Δ*rtxA::nptI* cartridge from the resulting construction (pKK1625) was liberated and ligated with SpeI-SphI-digested pDM4 (24) to generate pKK1628 (Table 1). *Escherichia coli* S17-1 λ*pir, tra* strain (22) containing pKK1628 was used as a conjugal donor to *V. vulnificus* MO6-24/O to generate the *rtxA* mutant KK1604 (Table 1). The conjugation and isolation of the transconjugants were conducted using a previously described method (27).

**Table 2:** Oligonucleotides used in this study.

**Name**	**Oligonucleotide sequence (5′ → 3′)^a,b^**	**Use**
**FOR MUTANT AND REVERTANT CONSTRUCTION**		
RTXA01-F	TGGAGTGCGTGAAATATCTCCATCC	Deletion of *rtxA* ORF, amplification of intact *rtxA*
RTXA01-R	TTCTGGATCCGACAGTGACATGATC	Deletion of *rtxA* ORF
RTXA02-F	TGTCGGATCCAGAAACGGGC	Deletion of *rtxA* ORF
RTXA02-R	ATTGGCTAGGTTTGCGGTGTGA	Deletion of *rtxA* ORF, amplification of intact *rtxA*

a *The oligonucleotides were designed using the V. vulnificus MO6-24/O genomic sequence (GenBank™ accession number CP002469 and CP002470, www.ncbi.nlm.nih.gov)*.

b *Regions of oligonucleotides not complementary to the corresponding genes are underlined*.

To complement the *rtxA* mutation, *rtxA* revertant was constructed as previously described (16). In brief, the region containing the intact sequences of *rtxA* was amplified by PCR using RTXA01-F and RTXA02-R as primers. The amplified intact *rtxA* was ligated into SpeI-SphI-digested pDM4 to generate pKK1629 (Table 1) and pKK1629 was transferred into the *rtxA* mutant KK1604 to generate the *rtxA* revertant KK1606 by conjugation.

### Preparation of murine bone-marrow-derived dendritic cells (BMDCs)

We generated BMDCs using the method originally described by Inaba et al. (28) with some modifications (8). In brief, the isolated bone-marrow cells were flushed out from the femurs and tibiae of mice, and red blood cells were depleted with RBC lysis buffer containing 0.15 M NH_4_Cl, 1 mM KHCO_3_, and 0.1 mM EDTA. For 7 days, cells (5 × 10^5^ cells/mL) were cultured in petri dishes with 10 mL of Roswell Park Memorial Institute (RPMI)-1640 medium (Thermo Fisher Scientific, Inc., Waltham, MA, USA) supplemented with 10% fetal bovine serum (FBS; Gibco; Thermo Fisher Scientific, Inc.), 2-mercaptoethanol (2-ME; 50 μM; Sigma-Aldrich; Merck Millipore, Darmstadt, Germany), 10 mM of HEPES, 100 U/mL of penicillin, and 0.1 mg/mL of streptomycin (Corning and Invitrogen; Thermo Fisher Scientific, Inc.) in the presence of granulocyte-macrophage colony-stimulating factor (GM-CSF; 10 ng/mL; ProSpec, Rehovort, Israel). At 3 and 5 days of culture, 5 mL of fresh medium containing 10 ng/mL of GM-CSF were added. The loosely adherent DCs were harvested on day 7 for subsequent experiments.

### *In vitro* infection protocol

Dendritic cells grown for 7 days were seeded onto a 24-well plate (1.5–2 × 10^6^ cells/mL) in an antibiotic-free growth medium. Before infection, the bacteria were centrifuged at 2,420 × *g* for 3 min at room temperature, resuspended, and adjusted to 1.5–2 × 10^8^ CFU/mL in antibiotic-free RPMI 1640 media. The DCs were infected with *V. vulnificus* WT and mutants at various multiplicities of infection (MOI; the ratio of bacteria number to BMDC number) for various durations. In particular, DCs were infected at an MOI of 1 for 30 or 60 min to evaluate the expression of surface markers and the secretion of cytokines, respectively or for 60 min at an MOI of 10 for co-culture with naïve CD4^+^ T cells. After infection, the cells were washed twice with PBS and incubated for 20 h in an antibiotic-containing growth medium at 37°C under 5% CO_2_.

### *In vitro* co-culture of CD4^+^ T cells with DCs

Naïve CD4^+^ T cells isolated from the axillary, lateral axillary, inguinal, popliteal, and submandibular lymph nodes of mice were purified using magnetic beads (MACS; Miltenyi Biotec GmbH, Bergisch Gladbach, Germany) and the purity of CD4^+^ T cells was confirmed to be >97% by flow cytometry. In a co-culture system, *V. vulnificus*-infected DCs (1 × 10^5^ cells/mL) were mixed with naive CD4^+^ T cells (5 × 10^5^ cells/mL) at a ratio of 1:5 in the presence of anti-CD3ε (1 μg/mL; cat. no. 553057) and CD28 (1 μg/mL; cat. no. 553294) monoclonal antibodies (mAbs) (BD Biosciences, San Diego, CA, USA). After 3 days, the cells were re-stimulated with 50 ng/mL of phorbol 12-myristate 13-acetate (PMA), 1 μg/mL of ionomycin, and 1 μL/mL of GolgiPlug for 6 h at 37°C and 5% CO_2_. After 6 h, the cells were harvested and stained with fluorescent antibodies for flow cytometric analysis.

### *In vivo* infection protocol and lamina propria cell isolation

To ablate normal flora, mice were given drinking water containing rifampicin (50 μg/mL) for 24 h. Before infection with bacteria, food and water were eliminated from the cages of mice to empty their stomachs for 12 h. In the subsequent step, 100 μL of bacterial suspension containing 1 × 10^7^ CFU of *V. vulnificus* was orally inoculated immediately following the administration of 50 μLof 8.5% (w/v) sodium bicarbonate (NaHCO_3)_. Mice were sacrificed at day 2 post-infection for the isolation of cells from the lamina propria of small intestines, as previously described (29). In brief, the small intestines from the mice were washed in cold PBS to clear feces. Fat tissues and Peyer's patches were removed and the intestines were longitudinally cut, followed by washing with cold PBS. The intestines were then cut into pieces (2–3 cm) and incubated in RPMI medium containing 1 mM ethylenediaminetetraacetic acid (EDTA) with gentle stirring at 37°C for 15 min, followed by washing with warm PBS. The incubation in EDTA-containing medium was performed twice. The tissues were subsequently finely cut and incubated in RPMI containing 0.1 mg/mL of collagenase D (Roche Diagnostics, Basel, Switzerland) at 37°C for 30 min with gentle stirring. The incubated supernatants were collected using a 70-μm cell strainer, and the unfractionated cells were centrifuged at 400 × *g* for 3 min at 4°C. The lamina propria lymphocytes were isolated with 40 and 85% Percoll gradient media (GE Healthcare Life Sciences, Little Chalfont, UK) by gradient centrifugation.

### Flow cytometric analysis

Antibodies used for both surface and intracellular staining were diluted at 1:250. The following mouse mAbs were used for flow cytometric analysis: CD4-FITC (cat. no. 553047), CD11c-FITC (cat. no. 553801), CD40-PE (cat. no. 553791), CD80-PE (cat. no. 553769), I-A^b^-PE (cat. no. 553552) (BD Biosciences, San Diego, CA, USA), IL-17A-APC (cat. no. 17-7177; eBioscience, Inc., San Diego, CA, USA). To detect intracellular IL-17A, the cells were re-stimulated for 6 h with 50 ng/ml PMA, 1 μg/ml ionomycin, and 1 μl/ml Golgiplug at 37°C and 5% CO_2_. Subsequently, the cells were intracellularly stained after permeabilization of the cells using Cytofix/Cytoperm kits (BD Biosciences, San Diego, CA, USA). The stained cells were observed using a flow cytometer (FACSCalibur or BD Accuri C6 Plus; BD Biosciences, San Diego, CA, USA) gated on live CD11c^+^ or CD4^+^ cells. The data were analyzed using CellQuest software version 4.0.2 (BD Biosciences, San Diego, CA, USA).

### Semi quantitative reverse transcription-polymerase chain reaction (RT-PCR) analysis

Total RNA obtained from the cells using TRIzol reagent was reverse transcribed into cDNA using a RocketScript™ Reverse Transcriptase kit (E-3162; Bioneer Corporation, Daejeon, Korea). PCR was conducted using 1 μl of each 5′ and 3′ primer, 1 μl of cDNA (50 ng). dH2O was then added to a final volume of 20 μl. PCR amplification of the cDNA was then performed using AccuPower^®;^ PCR PreMix (K-2016; Bioneer Corporation, Daejeon, Korea) with a thermal cycler (MJ Research, Inc., Watertown, MA, USA or Bioneer Corporation, Korea). The sequences of the PCR primers used in the present study were as follows: Murine IL-1β, forward 5′-CTAAAGTATGGGCTGGACTG-3′ and reverse 5′-AGCTTCAATGAAAGACCTCA-3′; murine IL-6, forward 5′-TGAACAACGATGATGCACTT-3′ and reverse 5′-CGTAGAGAACAACATAAGTC-3′; murine IL-23p19, forward 5′-AGCGGGACATATGAATCTAC-3′ and reverse 5′-TAAGCTGTTGGCACTAAGGG-3′; murine TGF-β, forward 5′-TATAGCAACAATTCCTGGCG-3′ and reverse 5′-TCCTAAAGTCAATGTACAGC-3′; murine β-actin, forward 5′-TGGAATCCTGTGGCATCCATGAAA-3′ and reverse 5′-TAAAACGCAGCTCAGTAACAGTCCG-3′. The temperature condition for PCR amplification was 95°C for 5 min; followed by 28–36 cycles consisting of 95°C for 30 s, 55–61°C for 30 s, and 72°C 30 s; plus a final cycle of 72°C for 5 min. The PCR reactions were performed for 28–36 cycles. After amplification, the products were separated on 1.5% (w/v) agarose gels stained with StainingSTAR (DyneBio, Gyeonggi-do, Korea).

### Cytokine assays

Dendritic cells were infected with *V. vulnificus* at the indicated MOIs and times, washed and seeded into a 96-well plate (2 × 10^4^ cells/well), followed by further incubation for 20 h. The cell supernatant was obtained to measure the levels of secreted cytokines. The quantities of IL-1β, IL-6, and IL-23 in the culture supernatants were determined using Mouse ELISA Ready-Set-Go! kits (IL-1β; eBioscience, Inc., San Diego, CA, USA) and a Mouse IL-6 ELISA kit (BD Biosciences, San Diego, CA, USA) or sandwich ELISA with anti-mouse IL-23p19 monoclonal antibody (clone 5B2) for plate coating and biotinylated anti-mouse IL-12/23 p40 monoclonal antibody (clone C17.8). A standard curve was generated using recombinant IL-23 (eBioscience, Inc., San Diego, CA, USA). The levels of secreted IL-17A in the culture supernatants were determined using a Mouse ELISA Ready-Set-Go! kit (IL-17A; eBioscience, Inc).

### Statistical analysis

Statistical analysis was performed with unpaired Student's *t*-test for pairwise comparisons or one-way analysis of variance (ANOVA) with a Bonferroni *t*-test for multiple comparisons in Sigmaplot version 12.5 (Systat Software Inc., Washington, USA). *P* < 0.05 was considered statistically significant.

## Results

### Infection with *rtxA* mutant strain of *V. vulnificus* affects DC maturation and activation

**Figure 1:**
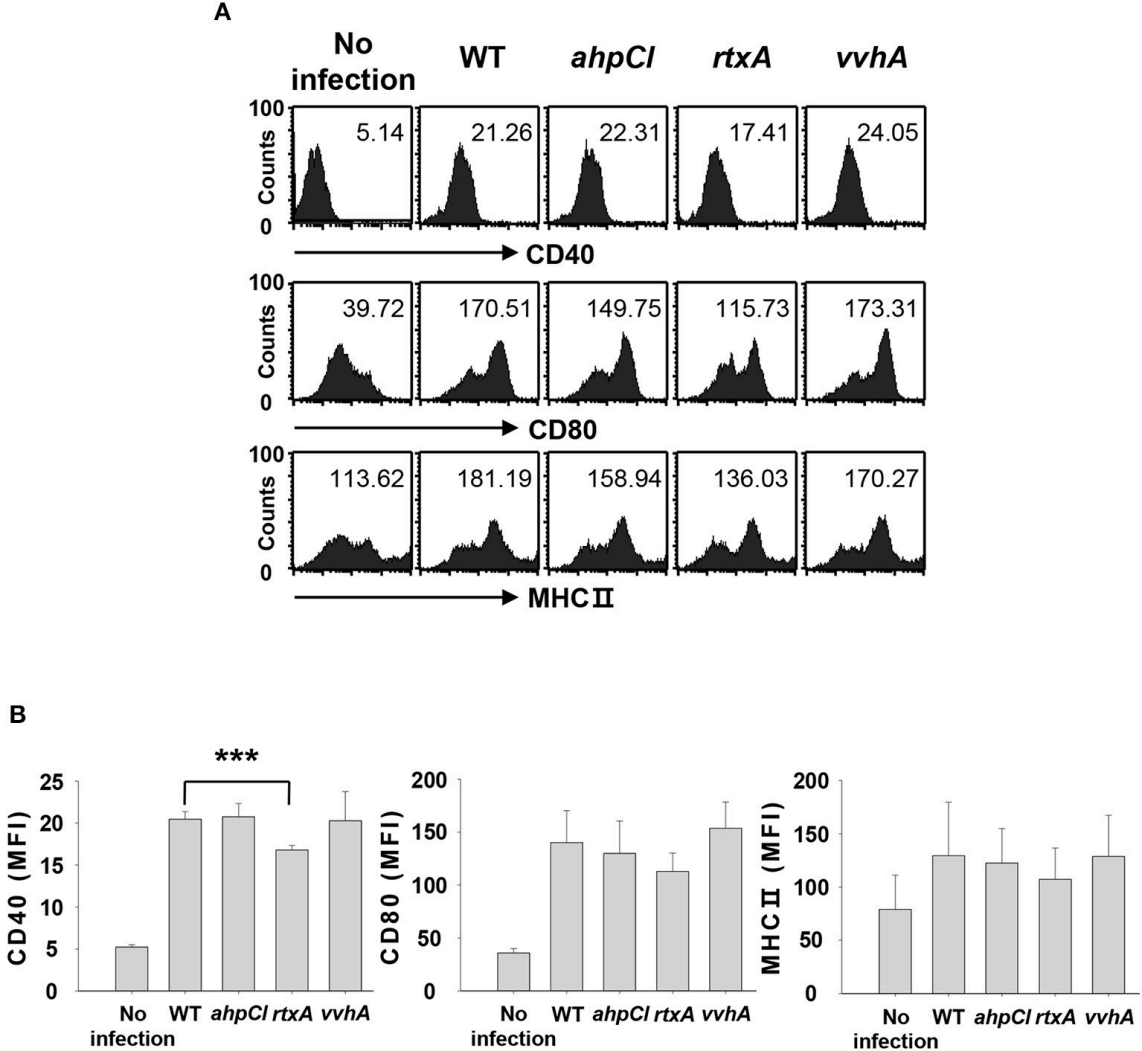
*Vibrio vulnificus rtxA* mutant was defective in inducing maturation and activation of DCs. **(A)** DCs were infected with WT and mutant *ahpCl, rtxA*, and *vvhA* mutant *Vibrio vulnificus* at an MOI of 1 for 30 min, followed by washing of cells with PBS and subsequent culturing for 20 h in the presence of antibiotics. After 20 h, the cells were stained with antibodies targeting CD40, CD80, and MHC II and their expressions were determined by flow cytometric analysis. The data shown in **(A)** are representative of three independent experiments, and bar graphs **(B)** represent the means ± SD of three independent experiments. ***p < 0.005 as determined by Student's *t*-test for pairwise comparisons. WT, wild type; *ahpCl, ahpCl* mutant; *rtxA, rtxA* mutant; *vvhA, vvhA* mutant.

We previously demonstrated that infection with WT *V. vulnificus* results in the induction of DC maturation and activation, leading to Th17 cell stimulation (8). Although several studies have identified virulence factors of *V. vulnificus*, the specific virulence factors involved in Th17 responses are unknown. We evaluated the abilities of several virulence factors from *V. vulnificus* to induce Th17 cell responses by infecting DCs at a multiplicity of infection (MOI) of 1 for 30 min with the WT strain and mutant strains with defects in *ahpCl, rtxA*, and *vvhA* genes encoding peroxiredoxin, RtxA, and hemolysin, respectively. The expression of maturation/activation-related cell surface markers, including CD40, CD80, and major histocompatibility complex (MHC) II, was analyzed with flow cytometry. As shown in Figures 1A,B, expression levels of these markers were reduced in *rtxA* mutant-infected DCs compared to those in DCs infected with the WT strain. In particular, CD40 expression level was significantly reduced in *rtxA* mutant-infected DCs. On the contrary, no differences in CD40 expression level were observed between DCs infected with *ahpCl* and *vvhA* mutant strains and WT-infected DCs. These data suggest that RtxA may act as a virulence factor of *V. vulnificus* that induces maturation and activation of DCs.

### DCs infected with *rtxA* mutant strain show defective secretion of Th17 polarization-related cytokines

**Figure 2:**
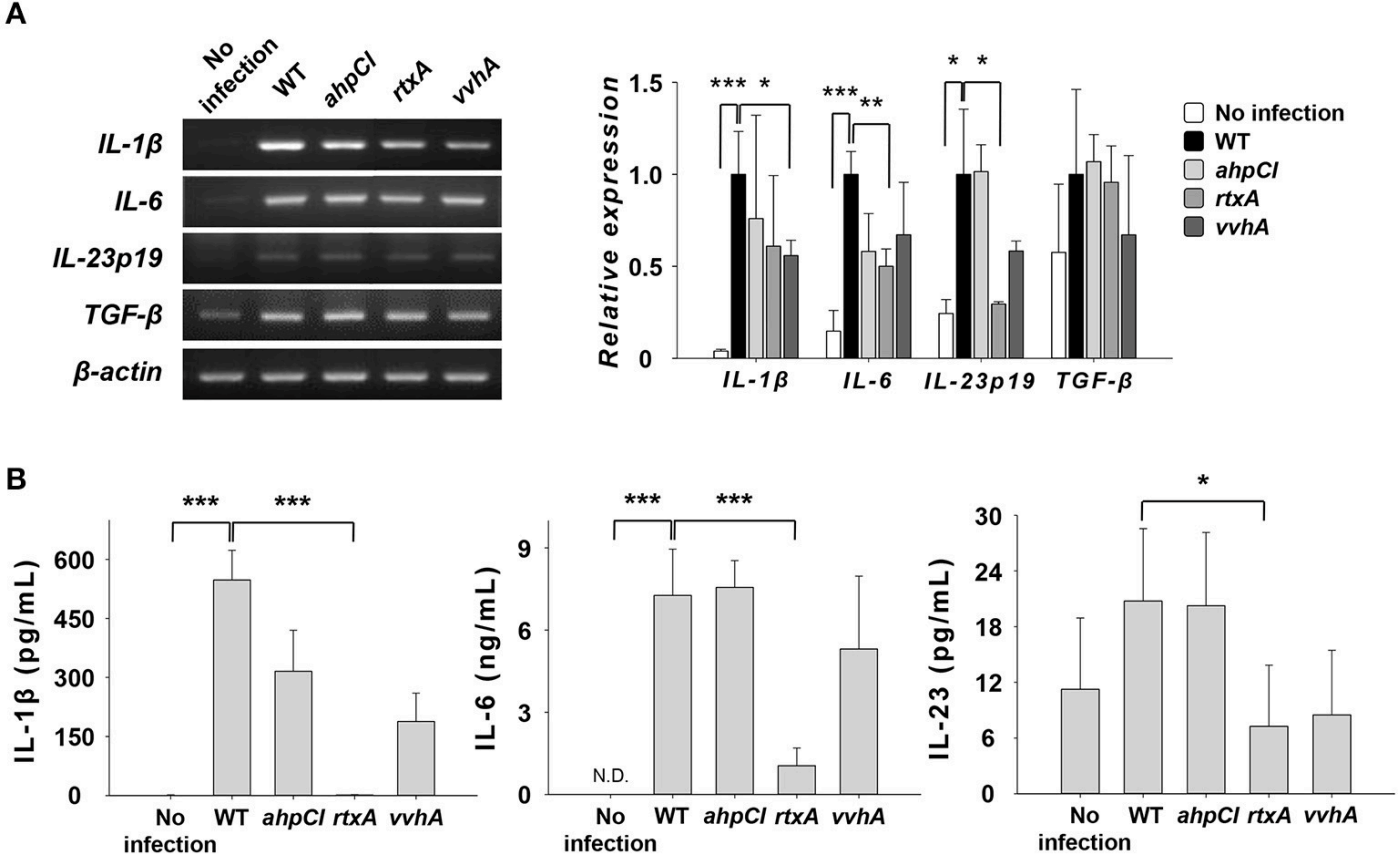
*Vibrio vulnificus rtxA* mutant was defective in inducing the secretion of Th17 polarization-inducing cytokines. **(A)** Total RNA was extracted from DCs infected with WT and mutant *ahpCl, rtxA*, and *vvhA* mutant *Vibrio vulnificus* at an MOI of 1 for 30 min. The expression levels of mRNAs for several Th17-polarizing cytokines were determined by RT-PCR. **(B)** DCs were infected for 60 min with WT and mutant *ahpCl, rtxA*, and *vvhA* mutant *Vibrio vulnificus* at an MOI of 1. The protein levels of IL-1β, IL-6, and IL-23 in the supernatants collected were determined by ELISA. The data shown in **(A)** are representative of three independent experiments, and bar graphs represent the means ± SD of three independent experiments. *p < 0.05, **p < 0.01, ***p < 0.005 as determined by Student's *t*-test for pairwise comparisons. WT, wild type; *ahpCl, ahpCl* mutant; *rtxA, rtxA* mutant; *vvhA, vvhA* mutant.

In our previous study, the expression and secretion of IL-1β and IL-6, the major Th17-polarizing cytokines, increased in DCs upon infection with the WT strain (8). To investigate the role of some virulence factors in the expression of cytokines related to Th17 cell induction, we infected DCs with WT and mutants of *V. vulnificus* at an MOI of 1 for 30 or 60 min and evaluated their expressions at the mRNA (Figure 2A) and protein (Figure 2B) levels. As shown in Figure 2A, the mRNA expressions of IL-6 and IL-23p19 were reduced in the *rtxA* mutant-infected group but not in cells infected with *ahpCl* and *vvhA* mutant strains, except for IL-1β expression in the *vvhA* mutant group. The secretion levels of IL-1β and IL-6 were significantly decreased in *rtxA* mutant-infected DCs compared to those in cells infected with other mutant strains (Figure 2B). In contrast, the expression level of transforming growth factor beta (TGF-β), a Th17 cell-related cytokine, was similar between mutant-infected and WT-infected DCs (Figure 2A). These results indicate that RtxA may be involved in Th17 cell induction caused by WT *V. vulnificus*.

### Induction of Th17 responses is reduced *in vitro* and *in vivo* after rtxA-deficient *V. vulnificus* infection

**Figure 3:**
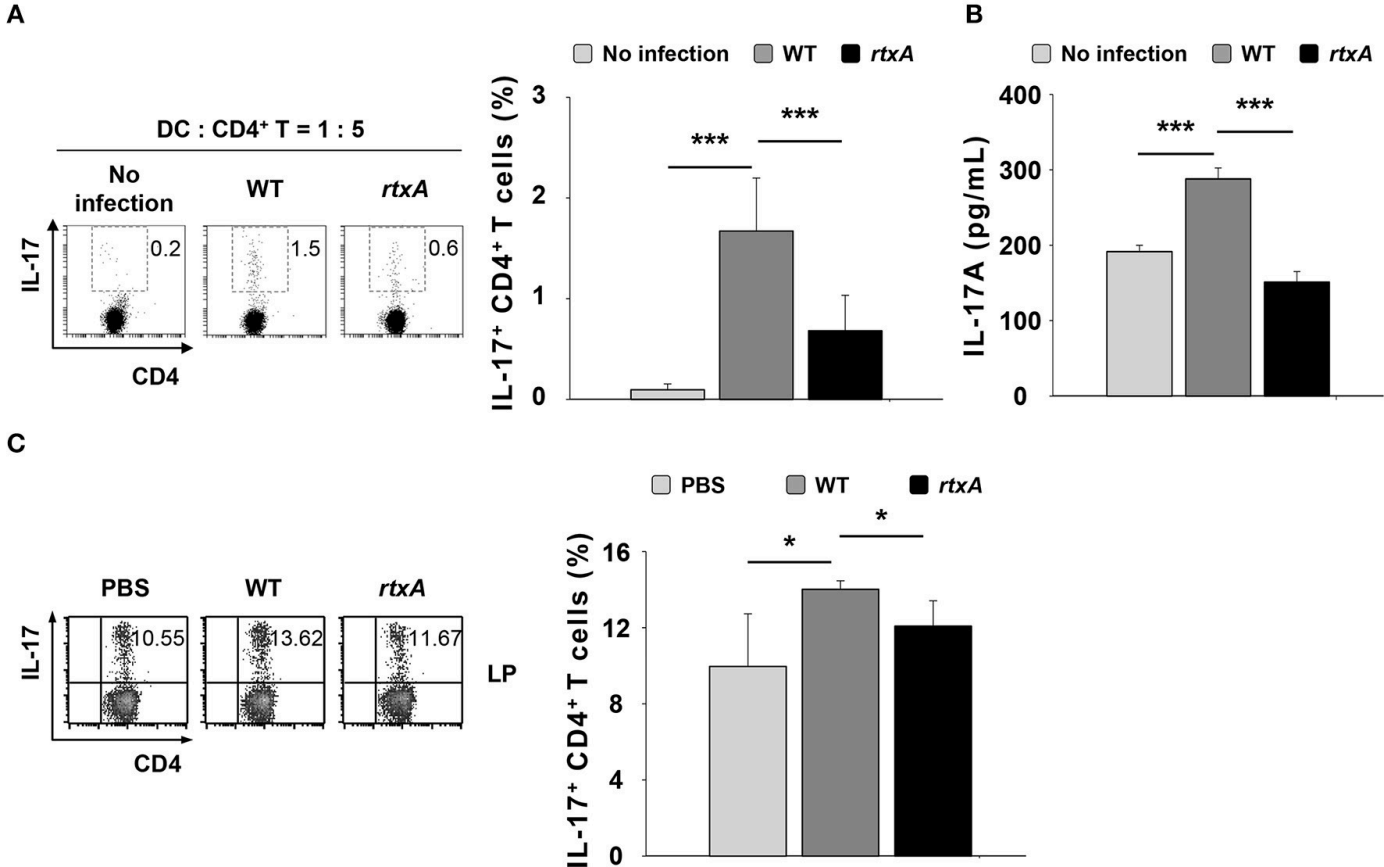
*Vibrio vulnificus rtxA* mutant was defective in inducing Th17 cell responses *in vitro* and *in vivo*. DCs were infected with WT and *rtxA* mutant *V. vulnificus* at an MOI of 10 for 60 min. After 20 h, DCs were co-cultured with naïve CD4^+^ T cells isolated from lymph nodes for 3 days in the presence of anti-CD3ε and anti-CD28 mAbs, followed by flow cytometric analysis of CD4 and IL-17 expression **(A)** or ELISA for IL-17A levels in the supernatants **(B)**. **(C)** The expression of CD4 and IL-17 in the lamina propria samples of uninfected mice or mice infected with WT and *rtxA* mutant (1 × 10^7^ CFU per mouse) were analyzed by flow cytometry. The data shown in **(A,C)** are representative of three independent experiments, and bar graphs represent the means ± SD of three independent experiments. Bars and error bars in **(B)** represent the mean ± SD of results performed in triplicate. *p < 0.05, ***p < 0.005 as determined by Student's *t*-test for pairwise comparisons. WT, wild type; *rtxA, rtxA* mutant.

Dendritic cells are antigen-presenting cells involved in the polarization of naïve CD4^+^ T cells into each subset such as Th1, Th2, and Th17, depending on the cytokines they produce (30). To determine whether DCs infected with the *rtxA* mutant strain have a weaker ability to induce Th17 cell responses, we infected DCs with the WT or *rtxA* mutant strain at an MOI of 10 for 60 min, and subsequently co-cultured these cells with naïve CD4^+^ T cells at a ratio of 1:5 (DC: CD4^+^ T). We evaluated the population of IL-17-expressing CD4^+^ T (Th17) by flow cytometry (Figure 3A) and the levels of secreted IL-17A in the supernatant by ELISA (Figure 3B). As shown in Figures 3A,B, WT-infected DCs displayed an increased proportion of Th17 cells and secretion of IL-17A as compared to the uninfected DCs, whereas the increased in Th17 cell responses and secretion of IL-17A observed following by WT infection were significantly reduced in DCs infected with the *rtxA* mutant strain. These observations indicate that the ability of *rtxA* mutant-infected DCs to induce Th17 cell responses was lower than that of DCs infected with WT *V. vulnificus*. Consistent with the *in vitro* result, we observed a decrease in the population of Th17 cells in the small intestinal lamina propria of mice treated with the *rtxA* mutant as compared with those treated with the WT strain (Figure 3B). These results suggest that RtxA may play a critical role in the induction of Th17 responses by *V. vulnificus*.

### *V. vulnificus rtxA* revertant infection recovers the reduced induction of Th17 responses after *rtxA* mutant infection

**Figure 4:**
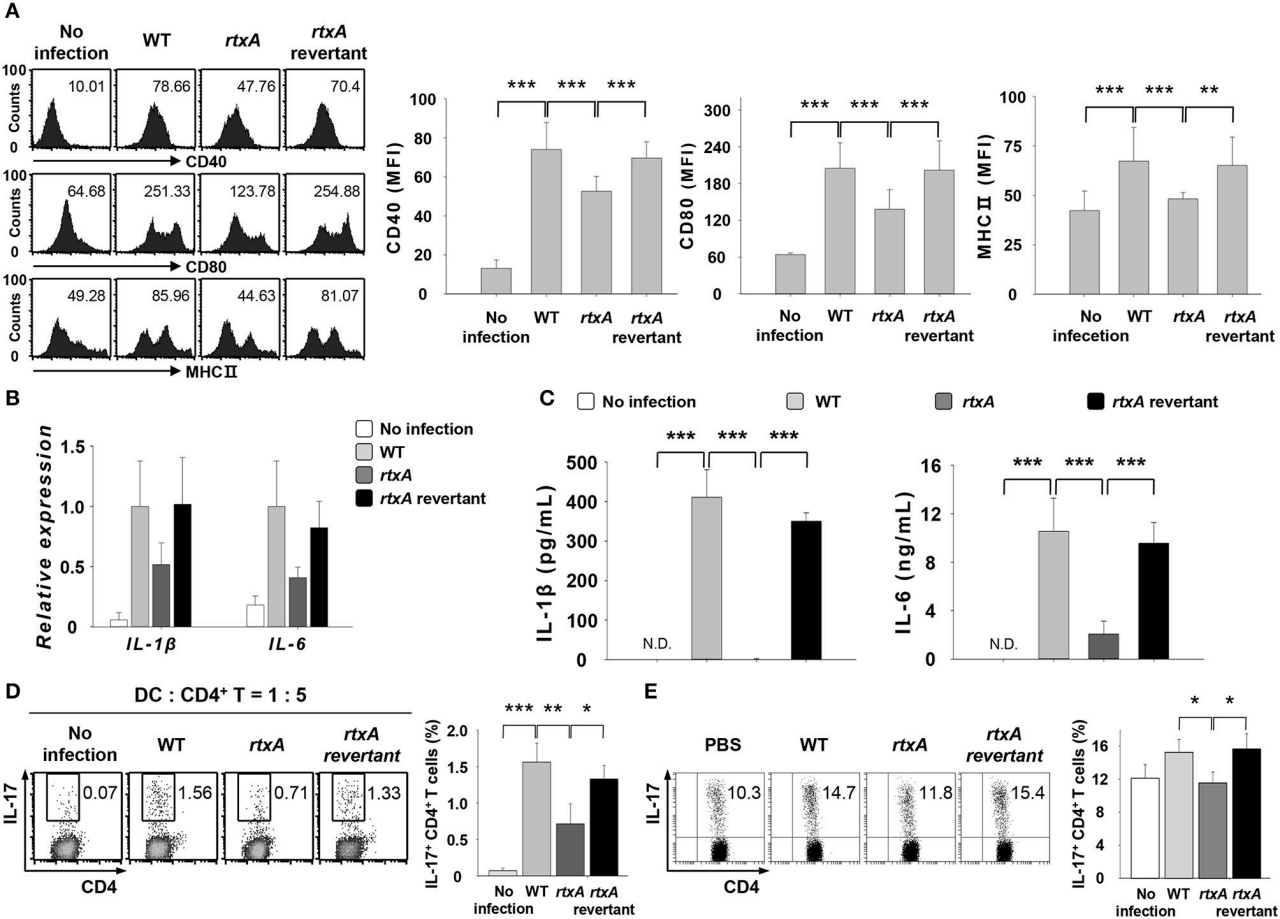
*Vibrio vulnificus rtxA* revertant restored the defect of the *rtxA* mutant by inducing the maturation and activation of DCs, secretion of Th17-polarizing cytokines, and Th17 cell responses. **(A)** DCs were infected with WT, *rtxA* mutant, and *rtxA* revertant *V. vulnificus* at an MOI of 1 for 30 min. **(B)** Total RNA was extracted from DCs infected with WT, *rtxA* mutant, and *rtxA* revertant strain. **(C)** DCs were infected for 60 min with *V. vulnificus* at an MOI of 1. **(D)** DCs were infected with WT, *rtxA* mutant, and *rtxA* revertant strain at an MOI of 10 for 60 min. After 20 h, DCs were co-cultured with naïve CD4^+^ T cells for 3 days in the presence of anti-CD3ε and anti-CD28 mAbs, followed by the flow cytometric analysis of CD4 and IL-17 expression. **(E)** The expression of CD4 and IL-17 in the lamina propria of uninfected mice or mice infected with WT, *rtxA* mutant*, and rtxA* revertant strains (1 × 10^7^ CFU per mouse) were analyzed by flow cytometry. The data shown in **(A,D,E)** are representative of three independent experiments, and bar graphs represent the means ± SD of three independent experiments. *p < 0.05, **p < 0.01, ***p < 0.005 as determined by one-way analysis of variance with a Bonferroni *t*-test for multiple comparisons. WT, wild type; *rtxA, rtxA* mutant.

To confirm the involvement of RtxA in the induction of Th17 responses *in vitro* and *in vivo*, we used the revertant strain of *rtxA* mutant. DCs were infected with *V. vulnificus* WT, *rtxA* mutant, and *rtxA* revertant at an MOI of 1 for 30 or 60 min or at an MOI of 10 for 60 min (Figures 4A–D). As shown in Figure 4A, the reduced expressions of maturation/activation-related surface markers in *rtxA* mutant-infected DCs were increased in *rtxA* revertant-infected DCs. Likewise, the reduced mRNA (Figure 4B) and protein (Figure 4C) expressions of IL-1β and IL-6 in *rtxA* mutant-infected DCs were restored in *rtxA* revertant-infected DCs to the levels observed in WT-infected DCs. Both *in vitro* (Figure 4D) and *in vivo* (Figure 4E), the percentages of IL-17^+^ CD4^+^ cells in the *rtxA* revertant-infected group were restored to the levels comparable with those observed in the WT-infected group. These results confirmed the crucial role of RtxA in the induction of Th17 responses against *V. vulnificus* infection.

### Infection with *V. vulnificus* mutant carrying the deletion of *hlyU*, an activator of *rtxA*, results in consequences similar to those observed with *rtxA* mutant infection

**Figure 5:**
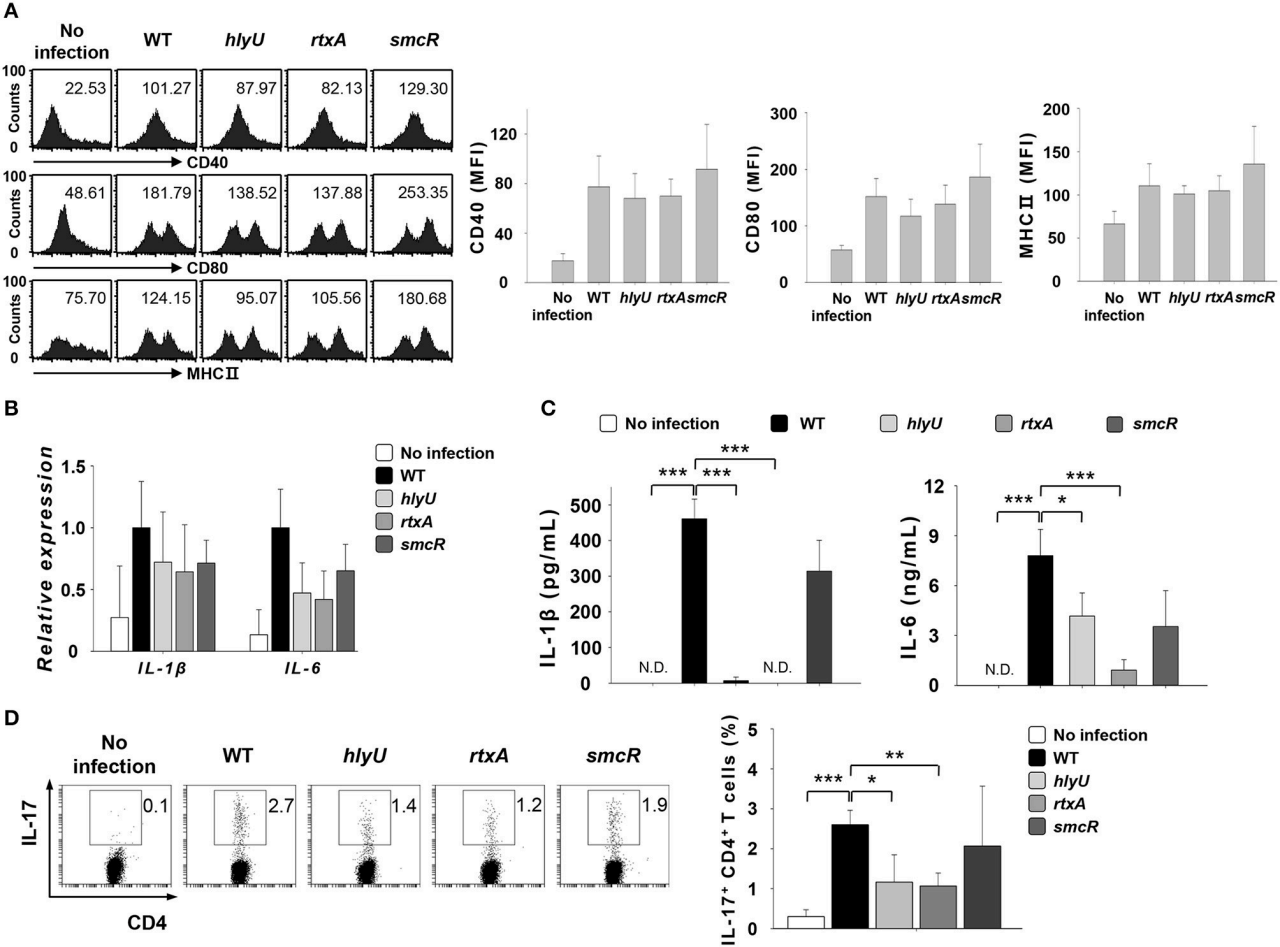
*Vibrio vulnificus hlyU* mutant reproduced the results observed for *rtxA* mutant. **(A)** DCs were infected with WT and *hlyU, rtxA, and smcR* mutant *V. vulnificus* at an MOI of 1 for 30 min. **(B)** Total RNA was extracted from DCs infected with WT and *hlyU, rtxA, and smcR* mutant strain. **(C)** DCs were infected for 60 min with WT and *hlyU, rtxA, and smcR* mutant strain at an MOI of 1. **(D)** DCs were infected with WT and *hlyU, rtxA, and smcR* mutant strain at an MOI of 10 for 60 min. After 20 h, DCs were co-cultured with naïve CD4^+^ T cells isolated from lymph nodes for 3 days in the presence of anti-CD3ε and anti-CD28 mAbs, followed by the flow cytometric analysis of CD4 and IL-17 expressions. The data shown in **(A,D)** are representative of three independent experiments, and bar graphs represent the means ± SD of three independent experiments. *p < 0.05, **p < 0.01, ***p < 0.005 as determined by Student's *t*-test for pairwise comparisons. WT, wild-type; *hlyU, hlyU* mutant; *rtxA, rtxA* mutant; *smcR, smcR* mutant.

HlyU positively regulates the expression of *rtxA* by interfering with H-NS, while SmcR acts as a repressor of *hlyU* (31–33). To test if the role of *rtxA* is regulated by HlyU, we infected DCs with *V. vulnificus* WT or mutant strains lacking the genes associated with *rtxA* gene expression, *hlyU and smcR* at an MOI of 1 for 30 or 60 min or at an MOI of 10 for 60 min (Figures 5A–D). As shown in Figure 5, infection with the *hlyU* mutant also resulted in decreased secretion of IL-1β and IL-6, the two Th17-polarizing cytokines (Figure 5C), although the decreases in the expression of DC surface markers (Figure 5A) and cytokine mRNAs (Figure 5B) were not significant. In addition, the differentiation of naïve CD4^+^ T cells into IL-17-secreting cells was reduced following *hlyU* mutant infection (Figure 5D). No significant difference was observed between *smcR* mutant-infected DCs and WT-infected DCs. Taken together, these results imply that RtxA confers *V. vulnificus* with an ability to promote Th17 responses *in vitro* and *in vivo*.

## Discussion

*V. vulnificus* inhabits marine environments and can invade the host through the ingestion of contaminated seafood or via an open wound. Upon entry, it can cause a wide range of diseases, which include gastroenteritis and primary sepsis (1), and activates the innate and adaptive immune responses via diverse virulence factors (8, 9). Many studies have identified several virulence factors involved in the innate immune response activation (14, 34–37). However, few studies have focused on the identification of the virulence factors that induce the adaptive immune response. Therefore, in the present study, we investigated the involvement of RtxA of *V. vulnificus* in the induction of Th17 cell responses and demonstrated that the increase in the population of Th17 cells was related to RtxA from *V. vulnificus*. Adenylate cyclase toxin (ACT) of *Bordetella pertussis*, a member of RTX toxin family belonging to a different subgroup, induces suppressive and modulatory effects on the immune response through the inhibition of the production of pro-inflammatory cytokines or induction of Th17 cells (38, 39).

Our previous findings demonstrated that *V. vulnificus* induced Th17 cell responses by up-regulating the secretion of IL-1β and IL-6 from DCs, and that the induction of IL-6 was sufficient and necessary for the increased Th17 cell responses (8). Presently, RtxA was demonstrated to be responsible for the secretion of the pro-inflammatory cytokines, IL-1β and IL-6, and consequent Th17 responses using *rtxA* mutant and *rtxA* revertant strains of *V. vulnificus. V. vulnificus* RtxA reportedly protects bacteria from phagocytosis (16). Therefore, it is conceivable that the *rtxA* mutant is phagocytosed and destroyed by DCs, while WT *V. vulnificus* carrying the intact *rtxA* gene may evade immune responses and induce DC activation and the production of pro-inflammatory cytokines, such as IL-1β and IL-6. Thus, *V. vulnificus* RtxA may be involved in the induction of Th17 responses. How RtxA of *V. vulnificus* promotes IL-1β secretion may involve the activation of the NLRP3 inflammasome by RtxA (14). Additionally, in the previous study, *B. pertussis* ACT enhanced the production of IL-6 in DCs in the presence of low concentrations of LPS (40). The RtxA of *V. vulnificus* likely acts synergistically with other virulence factors on IL-6 production.

*V. vulnificus* HlyU is a homolog for the hemolysin gene regulator of *V. cholerae* and regulates the expression of the *vvhA, vvpE*, and *rtxA* genes (31–33). To investigate the molecular mechanisms by which *rtxA* is regulated in *V. vulnificus*, DCs were infected with *hlyU* mutant. The lack of HlyU failed to increase the production of IL-1β and IL-6 from DCs. Eventually, Th17 cell responses were also down-regulated. These results clearly demonstrate that the Th17-inducing capacity of RtxA is regulated by HlyU. However, the decrease in the expression of DC surface markers and the transcriptional expression of IL-1β and IL-6 were not statistically significant. These observed discrepancies are possibly due to the other pathways that regulate the expression of *rtxA* and the function of *hlyU* gene, which is related to other virulence factors like *vvhA, vvpE*, and *smcR*. The deletion of SmcR, a negative regulator of HlyU, could not increase the secretion of IL-1β and IL-6 or Th17 responses. The absence of effect by *smcR* mutation may be due to the saturated function of HlyU in WT *V. vulnificus* or to other *rtxA* gene-activating pathways.

The expression of some virulence factors, including RtxA, increases upon the contact of *V. vulnificus* with host cells (41, 42). This observation may be associated with the results shown in Figure 1, wherein no significant decrease in the expression of CD80 and MHC class II surface molecules was observed in *rtxA* mutant-infected DCs, even though the secretion of Th17-polarizing cytokines and Th17 responses were significantly reduced in the *rtxA* mutant-infected group (Figures 2, 3). Thus, further studies are needed to identify additional virulence factors other than RtxA that are likely to be involved in the upregulation of DC surface molecules and/or Th17 responses. Similar to a previous study that showed the effects of secreted outer membrane vesicles of *V. cholerae* on DC activation, the release of Th17 polarization-related cytokines, and the induction of inflammatory Th17 cells (43), other structural components and/or secreted factors of *V. vulnificus* may also be involved in Th17 responses induction.

Previous studies reported that the exposure of the immune cells to sub-lytic concentrations of Panton-Valentine leukocidin, a pore-forming toxin of *Staphylococcus aureus*, resulted in immune cell activation and the release of pro-inflammatory cytokines, rather than cell lysis (44, 45). Furthermore, the amount of secreted RtxA upon contact with host cells increased as the degree of contact increased (46). Therefore, *V. vulnificus* RtxA may exert different effects on the host based on the nature of contact between *V. vulnificus* and the host cell. The close contact between the host cells and a large number of *V. vulnificus* results in the production of high (over lytic) concentrations of the RTX toxin, leading to host cell lysis and spread of infection into the bloodstream of the infected host. However, weak contact between the host cells and fewer *V. vulnificus* cells results in the production of low (sublytic) concentrations of *V. vulnificus* RTX toxin, leading to the induction of Th17 cell-mediated responses that are likely to be involved in controlling *V. vulnificus* infection. The RTX toxin of *V. vulnificus* forms pores on the host cell membrane and causes cell lysis (13, 15), and the expression of *rtxA* gene is induced after the contact with the host both *in vitro* (15) and *in vivo* (17). Th17 cells play an important role in maintaining mucosal barriers and contribute to pathogen clearance at mucosal surfaces (47), although their roles in *V. vulnificus* infection are unknown.

In summary, our study demonstrates that RtxA, one of the key virulence factors of *V. vulnificus*, induces the secretion of IL-1β and IL-6 from *V. vulnificus*-infected DCs and contributes to the induction of Th17 cells *in vitro*. In addition, RtxA increases the population of Th17 cells in the small intestinal lamina propria. Overall, these data establish the importance of RtxA in adaptive immune responses against *V. vulnificus*.

## Author contributions

AL participated in the design of the study, performed all of the experiments, the data collection, and the analysis, and drafted the manuscript. MK conceived and designed the experiments. DC contributed reagents, and materials. KJ and SC constructed and provided all *V. vulnificus* strains. TK conceived the study and participated in its design and the coordination and also performed the data analysis and writing of the manuscript, and has full access to all the data in this study with the financial support.

### Conflict of interest statement

The authors declare that the research was conducted in the absence of any commercial or financial relationships that could be construed as a potential conflict of interest.
